# Historically informed quality improvement: applying historical insights from Black women’s maternity care to contemporary health service redesign

**DOI:** 10.1093/intqhc/mzag113

**Published:** 2026-08-18

**Authors:** Jennifer Creese, Bharathy Kumaravel, Kellie Moss, Maxine Chapman, Natalie Darko, Floretta Cox, Ruw Abeyratne

**Affiliations:** School of Medical Sciences, University of Leicester, Leicester, United Kingdom; Greater Manchester Patient Safety Research Collaboration, University of Manchester, Manchester, United Kingdom; School of Medicine, University of Leicester, Leicester, United Kingdom; Public Health, Leicestershire County Council, Leicester, United Kingdom; Leicester Institute for Advanced Studies, University of Leicester, Leicester, United Kingdom; School of Healthcare, University of Leicester, Leicester, United Kingdom; School of Medical Sciences, University of Leicester, Leicester, United Kingdom; Leicester National Institute of Health Research (NIHR) Biomedical Research Centre, Leicester, United Kingdom; University Hospitals of Leicester NHS Trust, Leicester, United Kingdom; University Hospitals of Leicester NHS Trust, Leicester, United Kingdom

## Abstract

**Background:**

Quality improvement aims to systematically devise solutions to complex issues and problems in services and systems. However, many quality improvement initiatives aim to address outcomes rooted in historical policy and practice, where organizational memory may be long lost. A solid foundational understanding of past practice and policy is important to contextualize the present and highlight previous changes with sustained impact. However, it can also help to show deteriorating performance and suggest potential proven interventions which may be suitable to re-explore and identify areas which remain unchanged and problematic over the years, despite the best efforts of healthcare staff, leaders, and policymakers, indicating where deeper systemic and cultural change is required beyond the quality improvement approach.

**Methods:**

This paper introduces a tool and approach, the historically informed quality improvement (HIQI) matrix for mapping evidence around contemporary healthcare service quality against historical data on the same services to identify key areas for policy retention, intervention evaluation, quality improvement, and cultural change. The paper illustrates the tool’s use through a worked example of Black (African, African-Caribbean, and Mixed) ethnic minority women’s experiences in maternity care amid a backdrop of inequalities in maternity outcomes. Ten oral history interviews with Black women who used local hospital trust maternity services 1984–2004 were conducted, alongside focus groups with 11 Black women who had used the same services in the past 12 months.

**Results:**

In the worked example, using the HIQI tool identified that friendly and caring midwives were the heart of a positive birth experience; improvements in satisfaction were evident, resulting from proactive hiring of midwives from similar racial/ethnic groups to mothers, and increased attention to maternal mental health. However, connection to staff and time between appointments had deteriorated over the years, and women had less confidence in what to expect in pregnancy. Issues around racial and unconscious bias and lack of voice were a prominent theme across the years, suggesting these are deep-seated issues that require broader institutional and societal cultural change and are unreliable targets for quality improvement approaches. Three quality-improvement projects for the hospital trust were identified through the use of the HIQI tool.

**Conclusion:**

The HIQI approach detailed in this paper enables quality improvement practitioners to develop a richer understanding of changes to services over the years and develop evidence-based plans for quality improvement and service evaluation. The approach also highlights core issues which are deep-seated within the institution, requiring a comprehensive culture change strategy alongside quality improvement efforts.

## Introduction

Healthcare services are ever-evolving systems, as management teams seek to improve performance, safety and satisfaction year-on-year in the face of new, continuing or increasing demand. Healthcare quality improvement (QI) has arisen as a structured way of systematically devising solutions to bring about such improvements and address complex issues and problems in healthcare delivery [1]. However, QI initiatives are often held back by problems around the focus of improvement [2], availability of effective evidence of ‘what works’ [3], and a lack of understanding of the broader context into which they are developed and delivered [4]; not just the present context, but also its past.

To quote American literary great Maya Angelou, ‘You can’t really know where you are going until you know where you have been’ [5]. Current trends and issues in healthcare are rooted in historical realities, both at a societal level and at a local, organizational level. Without attention to the historical dimensions of healthcare, QI initiatives risk reinforcing rather than redressing long-standing inequities in access, experience, and outcomes. This historical grounding is particularly vital in addressing persistent inequalities in healthcare, as emphasized by Black feminist scholars who foreground/argue for the role of collective memory and lived experience in challenging dominant narratives and shaping emancipatory change [6].

A solid foundational understanding of the practice and policy of the past can help contextualize what is done (or not) in the present and why. However, the interventions and practices of the previous regime are oftentimes hard to concretely determine. Practice and policy documents identified as ‘outdated’ are often the first to be purged as new information technology or knowledge management systems are put in place; in the UK’s National Health Service (NHS), for example, most local operational policy and practice documents are kept on average for only 6 years [7]. Hard copies, if made, may be consigned to overstretched and under-resourced archives and records departments, where they become largely inaccessible. Unwritten knowledge of these previous practices and policies may reside in the memories of the staff involved in them; yet managers, on average, do not spend >5 years working in the same role, team, or organization [8], meaning that this tacit knowledge of the origins or contexts of practice and policy decisions may be lost [9]. For QI, this means that significant elements of the contextual understanding necessary for successful implementation may be missing and cannot inform initiatives.

It is well recognized that ‘it’s always been done this way’ is a dangerous and restrictive starting point for improvement. The historically informed quality improvement (HIQI) tool enables QI practitioners to answer the important question, ‘but, why?’. Shah and Sunol [10] call for the use of novel methodologies and ‘a range of designs, which are hitherto relatively underutilized in healthcare…in order to learn and adapt’ in QI. This paper addresses this gap by introducing a novel approach of drawing on the historical past to inform future QI. This paper introduces a tool and approach developed for mapping contemporary healthcare quality challenges against historical data on the same challenges to identify key areas for policy retention, intervention evaluation, QI, and cultural change. The use of this tool is illustrated with an example from a QI analysis of Black (African, African-Caribbean, and Mixed) ethnic minority women’s experiences in localized maternity care services.

## Materials and methods

### The HIQI matrix

The conceptual model presented in this study arose from planning discussions around the need for contextualizing historical research to deepen understanding for improvement in health care. With initial shared interests in maternal health inequalities, the team was multidisciplinary and cross-sectoral, comprising academics from anthropology (J.C.), history (K.M.), sociology and health sciences (N.D.), and nursing and midwifery (M.C.), joined by public health (B.K.) and maternity service (F.C.) clinicians and the local acute trust’s health equity lead (R.A.). Initially, the team, understanding that modern healthcare phenomena have historical antecedents [9], determined that developing historical insights into Black maternity experiences might develop enriched contextual information to inform local practice in order to address significant disparities in maternity outcomes and care satisfaction between Black and white mothers, as identified in local health data from Leicester, Leicestershire, and Rutland in the East Midlands region of the UK [11]. In the UK, Black mothers are 2.3 times more likely to die during pregnancy and childbirth than White mothers, 3.1 times more likely to suffer sepsis, and 3.8 times more likely to suffer a thrombosis, thromboembolism, or cardiac event [12]. Similar figures are also found in the USA [13] and among Indigenous Australians [14], and racism has been identified as a root cause of multiple inequalities in health, in maternity care, and beyond [15].

The team initially planned a free-standing oral history study of Black maternity experiences to shed light on the historical background to these disparities. However, a more explicit comparison between past experiences and present issues was needed to simplify the application of historical insights. While the study was being conducted, the first author developed a simple conceptual model designed to operationally apply historical contextual insight to contemporary activity for the purposes of QI. This model was iteratively revised and improved in discussion with the whole team until it reached the final form detailed here. The resulting tool would be able to be used for mapping historical and contemporary insights together for comparison, facilitating reflexive critique and allowing for more targeted QI and service evaluation development informed by both past and future contexts.

**Figure 1:**
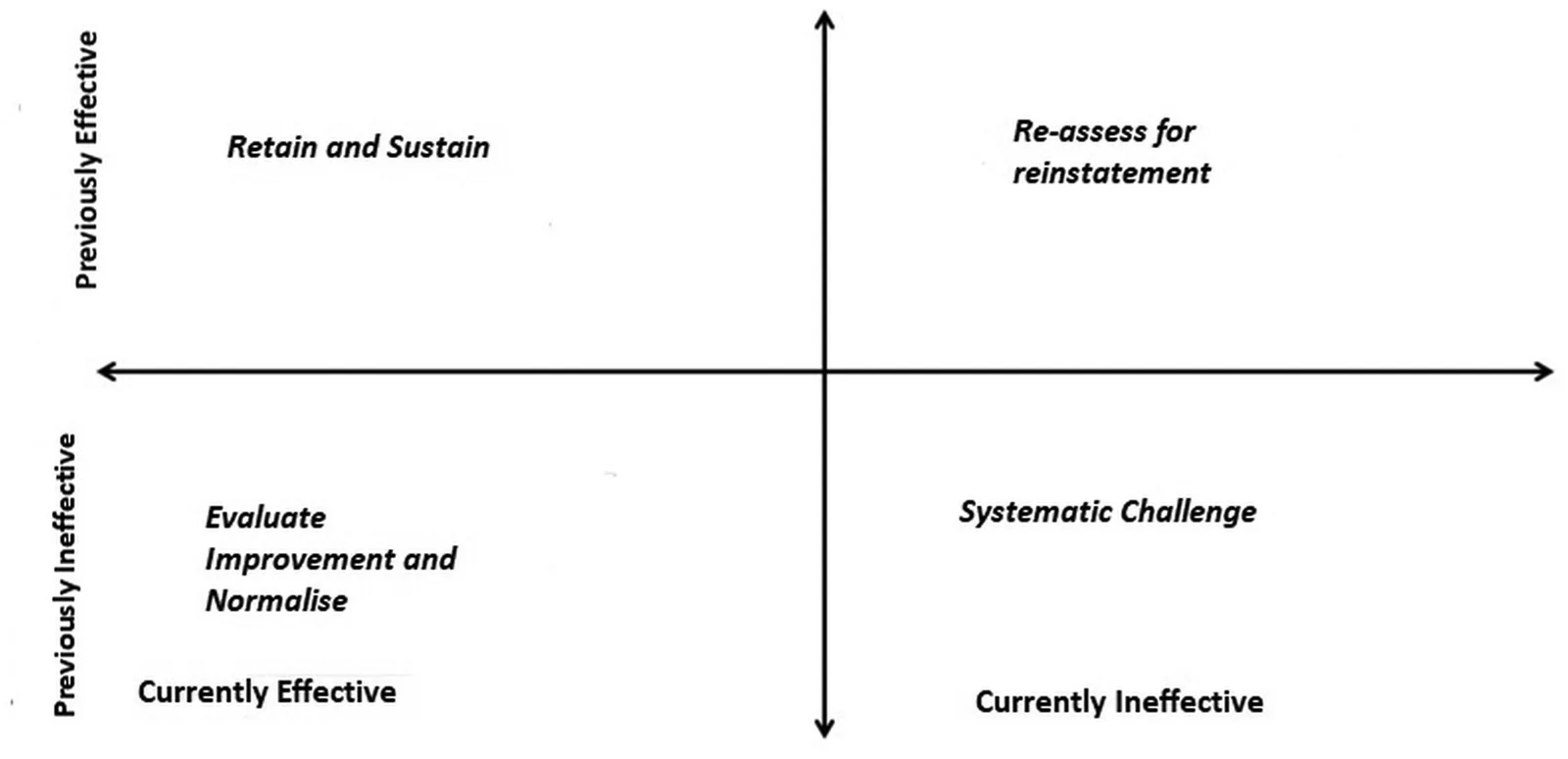
HIQI matrix. The four quadrants of the matrix allow the user to map whether the intervention or service in question was previously effective/ineffective against whether it is currently effective/ineffective. This helps identify appropriate strategies for quality improvement or assurance. Original design by the authors.

This tool, the HIQI matrix, allows researchers to graphically map out comparative insights into ‘what works’ and ‘what does not work’ within a service, with different areas of the matrix indicating different QI potentials and approaches (see Fig. 1). The HIQI matrix maps findings across two axes—what worked or did not work in the past and what works or does not work in the present. This presents four quadrants, each of which invites a different approach or challenge to the user.

Quadrant 1 (top left) contains elements which have continuously ‘worked’ or been judged positively or high-quality by stakeholders, both in the past and in the present. These elements are identified as the ‘heart of the service’; they are elements which should be the driving vision of the service against which future improvement should be benchmarked to ensure they are not compromised.

Quadrant 2 (bottom left) contains elements which were found to be negative or ‘didn’t work’ in the past, but have since been improved and now ‘work’ or are high quality; these elements are evidence of successful QI, but services need to ensure they are appropriately evaluated to determine enablers and factors which influence success in order to normalize them and more widely roll them out.

Quadrant 3 (top right) contains elements which were found to be positive or ‘worked’ in the past, but have since been lost or regressed; these are in many ways ‘prime candidates’ for QI, as there is evidence that they ‘worked’ before, but attention needs to be paid to examining contextual, institutional, or societal factors which changed and led to them ceasing in order to reintroduce them or use them as a basis for novel improvements.

Quadrant 4 (bottom right) contains elements that have always been problems, not ‘working’ in the past or in the current day; these elements demand a broader, expert-led approach to improvement, likely at a macro-system level, as they are linked to deep-seated cultural, systemic, or societal issues that are beyond the remit of local QI to fully address.

The following paragraphs show the use of the HIQI approach and matrix in practice, with a worked example from the research team’s exploration of Black women’s maternity experiences in both the past and the present. Comparative analysis through mapping to the HIQI matrix allowed a facilitated reflection on areas for QI, consolidation, and retention.

### Data collection

To generate historical and contemporary insights into disparities in maternity care, a qualitative approach was chosen for its capacity to offer a deeper understanding of the issue’s complexities than a survey could provide [16]. Interviews and focus groups were deemed the most appropriate methodologies [17] to understand women’s lived experiences and perspectives, which are central to meaning-making in qualitative and Black feminist research traditions [6].

To compile the historical dataset (Dataset 1), semi-structured interviews were conducted with Black women who had given birth under the care of maternity services in Leicester and Leicestershire. Participants self-identified as being of Black ethnicity and had given birth in the region between 1984 and 2004. This timeframe was selected to capture experiences from the previous generation of mothers, enabling comparison with more contemporary maternity care. Recruitment was conducted through social media, flyers in local African-Caribbean community facilities, and snowballing through personal networks, led by M.C., N.D., and F.C. Interviews were undertaken face-to-face or through MS Teams, at the discretion of the participant, by K.M. and M.C. All participants were provided with participant information sheets and gave their informed consent; the study maintained a feminist ethics of care [18] in managing potential distress on the part of participants and interviewers.

Independently, the contemporary dataset (Dataset 2) was collected using focus groups with Black mothers who were current or recent users of the same local maternity health services. Women who self-identified as being of Black ethnicity, who were pregnant or had given birth in the Leicester and Leicestershire region under the care of local health services within the last 12 months, were eligible for inclusion. Recruitment of a self-selecting sample of participants was conducted through flyers in local primary care and maternity care health services and community spaces. F.C. conducted two face-to-face focus groups and one online focus group. All participants were provided with information sheets and gave their informed consent, and a similar feminist ethics of care for participants was followed.

### Data analysis

Both datasets were analysed using reflexive thematic analysis [19]. Dataset 1 was initially analysed independently by three researchers (J.C., B.K., K.M.), with each analyst independently coding all transcripts. J.C. collated each analyst’s identified codes together and synthesized these into a single codebook and facilitated a collaborative process of reviewing and refining coding interpretations, in line with the reflexive approach discussed and agreed between analysts. This was then applied, reviewed, and final themes and subthemes were developed, in keeping with the collaborative thematic analysis approach of Richards and Hemphill [20]. N.D. reviewed theme refinement to support consistency and interpretive rigour. The same codebook was applied to the analysis of dataset 2, initially coded and summarized by F.C. and further analysed by J.C.

## Results

Ten women were interviewed in the historical study; 11 women participated in the focus groups of current-day mothers. Three themes were identified in the historical data, and corresponding data for each were found in the contemporary data. The theme ‘Mother-Midwife Relationships’ contained subthemes of trust, continuity, and expertise. The theme ‘Culture and Race’ contained subthemes of racism and hostility, cultural sensitivity and representation, and unconscious bias. The final theme ‘Information and Communication’ encompassed subthemes of speaking out, communicating, and being informed about what was going on. See Table 1 for themes and indicative quotes/data.

**Table 1:** Findings from oral history interviews and interviews with modern-day mothers.^a^

	Historical data		Contemporary data	
	Positive	Negative	Positive	Negative
Theme 1: Mother Midwife Relationships	Trust in health care provider ‘*My midwife was really nice…I could really open up to her*’		Good relationships with midwife/GP made positive experiences: ‘*I was going to go the GP every other day, my midwife was good to me, I had good reassurance*’.	Wanting continuity of care but not receiving it
	Continuity: ‘*When I walked through the door they recognised me, that was nice*’.		Staff asking about mental health or social concerns as well as medical	Too long between contact times with care staff: ‘*I couldn’t create a relationship with my midwife*’.
	Staff expertise: ‘*Our concerns were [eased] by the expertise of the staff, the way they handled everything*’.			Concerns about bad press over hospital being unsafe/staff not being skilled enough
	Staff taking time: ‘*medical team constantly checks upon you, they’ll call you, there is that love and concern coming from them*’.			
Theme 2: Culture and Race		Lack of cultural sensitivity: ‘*[midwives] did not respect my cultural beliefs, or practices*’.	Cultural and racial representation: ‘*Had a Black midwife in my labour, I felt happier and more comfortable*’.	Worried about racism and bias
		Hostility/Racism: *being called ‘that Black woman’, body shaming*		
		Unconscious bias: assumptions about women based on race/social standing		
Theme 3: Information and Communication	Information sharing: ‘*when I asked questions, I feel like things were explained*’.	Inability to speak out ‘*If I tried to draw attention to something they waved it off*’		Concerns about baby ignored or lacking empathy: ‘*[Staff] need to be a bit more caring and understanding when it comes to certain questions*’.
		Inability to communicate ‘*I was unable to tell her [midwife] things I was supposed to tell her’; ‘Would not turn to listen to what I had to say*’.		Lack of information, not knowing what to expect: ‘*I was new to the UK, it is difficult to navigate services, I didn’t feel I had enough information about my choices*’.

a Reflexive thematic analysis enabled identification of three cross-cutting themes: (i) Mother-Midwife Relationships, (ii) Culture and Race, and (iii) Information and Communication.

**Figure 2:**
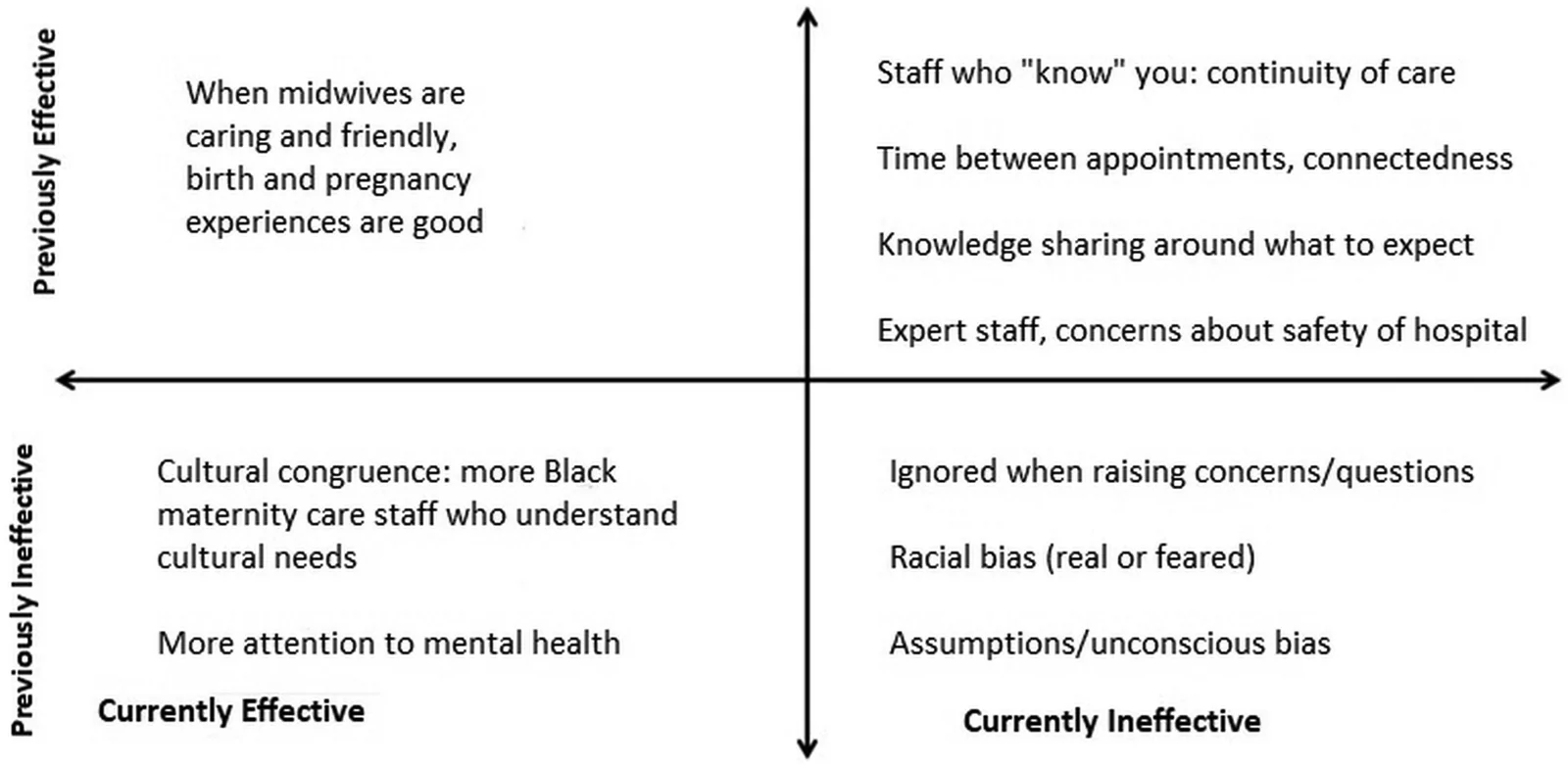
HIQI matrix completed for the case study example exploring factors influencing poor satisfaction and outcomes in maternity care among Black mothers in Leicester, UK. Caring, friendly midwives were identified as an area of both past and present strength. Continuity of care, appointment timing, knowledge sharing, and staff/hospital safety expertise were all identified as strengths of the past lost in the present. Cultural congruence and attention to mental health were identified as modern improvement. Patient lack of voice, racial bias, and unconscious bias were consistent areas of weakness across time.

Using the HIQI matrix, the themes and findings identified across both datasets were mapped out as to whether they worked/did not work and were past or present. The resulting completed matrix (see Fig. 2) could be used to determine areas which were good and needed to be kept, areas which were improved and needed evaluation and normalization, areas which had regressed and could be re-examined in a modern context, and deeper-seated areas which required systemic cultural change.

Based on this mapping exercise, the local NHS Trust was able to set priorities for service evaluation and improvement. In discussion with the Trust’s Director of Health Equality and Inclusion (R.A.), three priorities for QI were selected for scoping in the following year: improving continuity of care by matching mothers with nominated midwife-led care teams; improving connectedness between appointments with an online chat service and improving culturally tailored information on what to expect from pregnancy and maternity services with an online repository and app. The team also recommended an audit of hiring practices in the midwifery team to examine ethnic and racial diversity within the midwife workforce, and normalization of psychological health checks in antenatal care. The deeper issues around racial bias, unconscious bias, and lack of voice for Black mothers were highlighted as problems, but rather than solely being addressed through a hyper-local QI approach, it was acknowledged that these issues were part of a broader culture within the Trust, the NHS, and public institutions as a whole and needed to be addressed at a higher level organizationally, regionally, and nationally as part of an overarching culture change strategy.

## Discussion

### Statement of principal findings

Using the HIQI tool in this worked example, it was possible to gather insights from Black women as service users both past and present, compare them, and identify areas of continuous quality, improved service, deteriorated service, and deep-seated ­institutional/systemic problems.

### Interpretation within the context of the wider literature

In the worked example from Black maternity care, HIQI was able to identify relational aspects as the ‘gold standard’ of positive maternity care, echoing findings from other studies [21, 22] and reinforcing the importance of the interpersonal as a guiding principle in service delivery and improvement. The identification of a biopsychosocial approach as important in modern maternity care also aligns with findings from other studies [23], though the evidence for cultural congruence (i.e. mother and midwife coming from the same ethnic background) in other studies is more mixed [24]. Studies also point to modern-day issues around maintaining continuity of care [25] and information availability [26] as issues for improvement in maternity services internationally. This suggests that the issues identified by the HIQI approach as positive features or areas in need of improvement have a valid basis from which to work and reinforces that HIQI has high utility for facilitating effective QI planning.

### Implications for policy, practice and research

The HIQI approach presents QI practitioners with a practical tool for use in exploring service context and identifying areas for service evaluation and improvement. Context is key in QI, and the context of the past is important for benchmarking whether services have improved or declined, but these insights can be hard to articulate. The HIQI matrix provides a framework for this articulation in a way that can focus contextual understanding and target key areas of need. QI practitioners can use the HIQI approach in the initial stages of improvement planning and scoping; for example, the HIQI approach aligns well as a subset of existing QI models or schema. The approach fits into the problem definition activity of many models, such as the Definition step of the Six Sigma DMAIC methodology [27]. HIQI also aligns well with the iterative approach of PDSA, as historical insights contribute to the initial plan stage, and then insights generated at the study stage become future iterations’ historical insights for a later new plan stage [28] An additional advantage of this history-informed approach, particularly relevant in the public and community health space, is the opportunity to better develop community partnerships and relations through the process of listening to the stories of elders and leaders and engaging lived memory in service improvement [29] which makes it more likely to be sustainable [30]. This may equally apply to engaging staff, service users, community leaders, and other stakeholders.

With regard to methodology and evidence generation, in the worked example the team used more formal scholarly methods of oral history interviews, focus groups and reflexive thematic analysis. However, the HIQI approach is method-agnostic and can be used with any type of data as long as there is a combination of historical and contemporary insights to enable a longitudinal reflection on the service and inform QI. Questionnaires, forums, patient records, or internal service reports could all equally be used as sources of evidence for a HIQI analysis using the matrix. Likewise, complex analysis is not required—simple descriptive statistics or content analysis can be used to generate insights.

### Strengths and limitations

The HIQI approach provides a formalized way to draw on the past to understand and contextualize the present within a service that has an identified need to improve. Use of the matrix tool is simple and provides a concrete way to map insights and findings, accessible by novice QI practitioners and experts alike. The historically informed approach can help health services systematically map out where a service has been to better understand where it is now and draw on institutional and communal memory to consider improvement with a long-term rather than short-term view.

There are some inherent limitations to the use of the HIQI approach. Firstly, the use of historical data, whether collected through oral history or retrospective surveys, may run the risk of recall bias, where participants’ memories may be shaped by other stimuli including modern-day issues; triangulating with other sources of data such as archives or historical patient records can increase the rigour and reliability of insights [31]. Secondly, while the tool can identify initiatives and interventions that used to work well in the past, it requires broader critical and contextual thinking and information-gathering to establish why these initiatives were discontinued and whether they are applicable to the modern-day institution [32]. Thirdly, the matrix may, by identifying long-running problems as outside the scope of QI intervention, run the risk of making issues which require cultural change as ‘un-improvable’, pushing the will to solve them ‘down the road’. Care needs to be taken to ensure these broader cultural issues, such as safety, inclusion, and equity, underpin all improvement efforts and act as a lever for QI even if not an improvement target in themselves [33]. Local QI in these difficult and systemic areas can also be important for demonstrating the case for change in policy (local, regional and national) and highlighting good practice and potential impact. So, while institutional and system change is needed, QI may provide a ‘grass-roots’ movement and call to action to aid this.

A final consideration is with regard to representativeness. As shown in the worked example, participating mothers were from a range of ethnic and cultural backgrounds within the broad categorization of ‘Black’, including Black African, Black Caribbean, mixed heritage, and other Black identities. These intersecting and varied social, cultural, and care experiences may shape how maternity care is perceived and experienced, and one group’s experience is likely to be very different to any other’s. As such, the worked example findings, but equally any community-focused QI work, should be interpreted with consideration of this diversity when applying insights to inform equity-focused QI efforts.

## Conclusions

QI is complex and highly context-dependent; the HIQI approach detailed in this paper provides a way to contextualize and compare the progression of services and initiatives to better understand where changes have come from and what has worked (or not) and why. It can help to identify areas that have improved with a longer-term view, consider previously effective initiatives in a new light, and enable more successful QI by differentiating between service quality and service culture. This has great potential to become part of broader improvement methodology, allowing a systematic approach to developing contextual knowledge and engaging with others to promote co-developed and co-delivered service improvement.

## Data Availability

Data available upon reasonable request.
